# Motor imagery brain–computer interface rehabilitation system enhances upper limb performance and improves brain activity in stroke patients: A clinical study

**DOI:** 10.3389/fnhum.2023.1117670

**Published:** 2023-03-14

**Authors:** Wenzhe Liao, Jiahao Li, Xuesong Zhang, Chen Li

**Affiliations:** ^1^State Key Laboratory of Reliability and Intelligence of Electrical Equipment, School of Artificial Intelligence, Hebei University of Technology, Tianjin, China; ^2^Department of Neurosurgery, The Second Hospital of Hebei Medical University, Shijiazhuang, Hebei, China; ^3^Tianjin Key Laboratory of Environment, Nutrition and Public Health, Department of Occupational and Environmental Health, School of Public Health, Tianjin Medical University, Tianjin, China

**Keywords:** MI-BCI, upper limb, motor function rehabilitation, stroke rehabilitation, incurable patient

## Abstract

This study compared the efficacy of Motor Imagery brain-computer interface (MI-BCI) combined with physiotherapy and physiotherapy alone in ischemic stroke before and after rehabilitation training. We wanted to explore whether the rehabilitation effect of MI-BCI is affected by the severity of the patient’s condition and whether MI-BCI was effective for all patients. Forty hospitalized patients with ischemic stroke with motor deficits participated in this study. The patients were divided into MI and control groups. Functional assessments were performed before and after rehabilitation training. The Fugl-Meyer Assessment (FMA) was used as the primary outcome measure, and its shoulder and elbow scores and wrist scores served as secondary outcome measures. The motor assessment scale (MAS) was used to assess motor function recovery. We used non-contrast CT (NCCT) to investigate the influence of different types of middle cerebral artery high-density signs on the prognosis of ischemic stroke. Brain topographic maps can directly reflect the neural activity of the brain, so we used them to detect changes in brain function and brain topological power response after stroke. Compared the MI group and control group after rehabilitation training, better functional outcome was observed after MI-BCI rehabilitation, including a significantly higher probability of achieving a relevant increase in the Total FMA scores (MI = 16.70 ± 12.79, control = 5.34 ± 10.48), FMA shoulder and elbow scores (MI = 12.56 ± 6.37, control = 2.45 ± 7.91), FMA wrist scores (MI = 11.01 ± 3.48, control = 3.36 ± 5.79), the MAS scores (MI = 3.62 ± 2.48, control = 1.85 ± 2.89), the NCCT (MI = 21.94 ± 2.37, control = 17.86 ± 3.55). The findings demonstrate that MI-BCI rehabilitation training could more effectively improve motor function after upper limb motor dysfunction after stroke compared with routine rehabilitation training, which verifies the feasibility of active induction of neural rehabilitation. The severity of the patient’s condition may affect the rehabilitation effect of the MI-BCI system.

## 1. Introduction

Ischemic stroke refers to the necrosis of brain tissue caused by stenosis or occlusion of the feeding arteries of the brain (carotid artery and vertebral artery) and insufficient blood supply to the brain. Brain tissue damage leads to the rupture of the brain nerve pathways that control movement, language, and other functions, resulting in hemiplegia and even disability. According to the World Health Organization, stroke is the main cause of disability and mortality in China. In 2019, the number of stroke deaths reached 2.19 million, and there were 3.94 million new stroke cases. The number of stroke patients in China has maintained a sharp upward trend in recent years (China National Health Commission, 2021). Stroke patients have difficulty taking care of themselves because of motor dysfunction, which seriously affects their physical and mental health (Lee et al., 2021). Recovery of motor function in stroke patients has become an important topic in the field of rehabilitation. BCI methods are among the most effective tools for the design of rehabilitation systems. Quantitative electroencephalography (QEEG) can assist in detecting or predicting stroke evolution of stroke (Simon et al., 2007). An electroencephalogram (EEG) can assess the severity of stroke and can be used as a supplementary tool in the monitoring of acute ischemic stroke (Ajčević et al., 2021a). Non-invasive EEG can use the neurophysiological biomarkers obtained in the early stage before treatment as a prognostic parameter, which can help improve rehabilitation treatment (Ajčević et al., 2021b). With advances in brain science and computer technology, many BCI studies have focused on decoding EEG signals associated with whole-body kinematics/kinetics, motor imagery, various senses, and neuroprosthetic and neurorehabilitation devices (Abiri et al., 2019). Virtual reality (VR) can increase the efficiency of BCI rehabilitation systems. Functional electrical stimulation, robotic assistance, and Hybrid VR based models are the main BCI approaches for designing stroke rehabilitation systems (Muhammad et al., 2020). Stroke rehabilitation is based on plasticity of the nervous system. Repeated feedback stimulation during learning and training can strengthen the connections between neuronal synapses, thus helping to gradually repair, compensate, and reconstruct the cerebral cortex and other rehabilitation effects (Morioka et al., 2018). The MI-BCI is an active interaction mode (Jessica et al., 2018). When the human body performs motion imagination but the real limb has no obvious action, the sensorimotor cortex of the brain is in an active state (Alexandra et al., 2021). Compared with passive stimulation, active participation in the imagination process has a stronger inducing effect on the plasticity of the central nervous system. MI-BCI can promote reorganization of damaged brain regions and neural pathways by recruiting and enhancing the activity of undamaged neurons. Motor imagery has the potential to induce plasticity in the brain cells. It can accelerate the repair of neural functional connections between the external limbs and brain. The motor imaging brain–computer interface (MI-BCI) based on motor imagination converts the neural activity signals of the brain into control signals of computers or external devices. It can not only help people with limb movement disorders effectively control external devices but also provide a new strategy for rehabilitation treatment of stroke patients (Ana et al., 2021). The use of BCIs has significant immediate effects on the improvement of hemiparetic upper extremity function in patients after stroke, however, the limited number of studies does not support its long-term effects. BCIs combined with functional electrical stimulation may be better for functional recovery than other types of neural feedback (Bai et al., 2020). BCI–FES rehabilitation training can induce clinically significant improvements in motor function in chronic stroke patients. It can improve the functional integration and separation of brain networks and boost compensatory activity in the contralesional hemisphere to a certain extent (Zhan et al., 2022). MI-BCI can not only help patients with Limb Dyskinesia to effectively control external devices but also provide a new strategy for rehabilitation treatment (Kamal et al., 2021).

In this study, we designed an MI-BCI rehabilitation application system using EEG and visual, auditory, and electromyographic stimulation feedback. Forty stroke patients will be randomly divided into MI and control groups. The MI group will receive routine treatment and MI-BCI treatment, whereas the control group will only receive routine treatment. The feasibility and effectiveness of the MI-BCI system in the upper limb rehabilitation of stroke patients were verified by comparing the clinical scale, NCCT, and EEG between the two groups. By comparing the rehabilitation of motor function of patients in the MI group before and after treatment, we preliminarily explored whether the severity of the disease affects the use of MI-BCI and whether MI-BCI can be used by all patients.

## 2. Materials and methods

### 2.1. General information

During the rehabilitation process, patients with stroke were selected from a hospital between February 2019 and May 2020. (1) Inclusion criteria: ➀ those who met the diagnostic criteria of the Chinese guidelines for the diagnosis and treatment of acute ischemic stroke 2019 (formulated and issued by the cerebrovascular disease group of the neurology branch of the Chinese Medical Association) and had upper limb dysfunction and were responsible lesions; ➁ The patient had the first onset and was in the recovery period of ischemic stroke; ➂ Age 35–70 years old, without obvious cognitive dysfunction and in good mental state; ➃ Before the clinical trial, they did not receive formal motor imagery therapy; (2) Exclusion criteria: ➀ in the first aid, the patient is not out of danger and the vital signs are not stable; ➁ Having various primary malignant tumors, serious heart, liver, lung, kidney, endocrine, hematopoietic, immune system and other serious diseases (including but not limited to tumor, heart, lung, endocrine, blood and immune system diseases); ➂ The patient has arthritis, rheumatoid, fracture and other diseases or symptoms that affect the motor function of the upper limb; ➃ Patients who are mentally abnormal or relatively resistant to treatment; ➄ Patients or their family members cannot sign the informed consent form and cannot cooperate to insist on treatment.

First, the data were collected from 47 patients. During the initial MI-BCI data collection process, three patients were excluded because the accuracy of EEG collection was lower than the standard, and four patients voluntarily withdrew for personal reasons. According to the random distribution method and personal will, the patients were divided into the MI group (20 cases) and the control group (20 cases) (and the other 7 cases were added to the control group with the consent of themselves and their families, and they were given routine medical rehabilitation treatment instead of EEG-related rehabilitation training). Finally, a total of 20 stroke patients participated in the entire MI-BCI upper-limb rehabilitation training and voluntarily participated in the trial. They or their families signed an informed consent form and were able to adhere to treatment and examination. This study was reviewed and approved by the hospital ethics committee (approval number: Hebuthmec 2019002). The purpose of the experiment was to perform scene EEG acquisition and upper-limb rehabilitation training for stroke patients. This is a non-invasive experiment that will not cause harm to subjects and protect patients’ privacy. After initial treatment, patients regain basic mobility and then undergo rehabilitation in community hospitals; therefore, follow-up after 3–6 months is difficult to perform. The project team collected data before and after rehabilitation in a stroke hospital.

### 2.2. Research methods

All treatments in the MI and control groups were performed in accordance with “Chinese Guidelines for Stroke Prevention and Treatment (2021 edition).” The entire rehabilitation system consists of a mobile portable host, 64 channel EEG systems, myoelectric stimulation system, and motion imagination software. The repair of upper-limb motor neuron injury is completed through two categories of motor imagination. The patient imagines left- or right-hand movements, and the EEG amplifier transmits the patient’s EEG to the BCI online system. The online system analyses the EEG to obtain an output command, and the functional myoelectric stimulation equipment stimulates the limbs to provide feedback to the nervous system. Another external nerve pathway was built through the MI-BCI and myoelectric stimulation systems.

**FIGURE 1:**
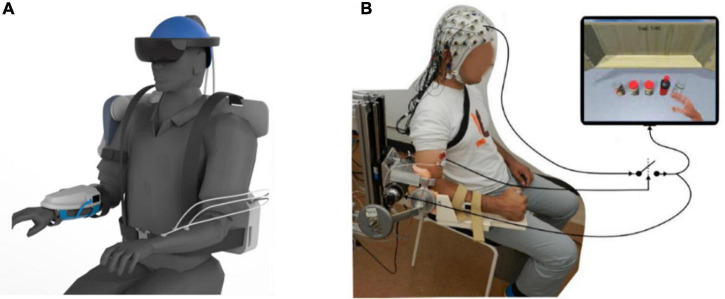
Motor imagery brain-computer interface (MI-BCI) upper limb rehabilitation system. **(A)** Ideal MI-BCI system. **(B)** Actual usage diagram.

Data Collection and Preprocessing: As shown in Figure 1, the EEG data were collected from 64 electrodes positioned according to the international extended 10–20 system. In the evaluation process, the EEG data was selected from 28 (“FPZ,” “FZ,” “FCZ,” “CZ,” “TP7,” “CP5,” “CP3,” “CP1,” “CPZ,” “CP2,” “CP4,” “CP6,” “P7,” “P5,” “P3,” “P1,” “PZ,” “P2,” “P4,” “P6,” “P8,” “PO7,” “POZ,” “PO4,” “PO8,” “O1,” “OZ,” “O2”) electrodes. EEG signals were sampled at 1000 Hz (SynAmps2, Neuroscan, USA). All 28 channels were grounded between the Fpz and Fz channels, and referenced to the binaural mastoid.

Surface EMG data were recorded using Pico EMG sensors (Cometa Italy2) from 16 muscles collected in a bipolar fashion: extensor digitorum, flexor digitorum superficialis, lateral head of the triceps muscle, long head of the biceps brachii muscle, pectoralis major, lateral deltoid, anterior deltoid, and upper trapezius on both sides. The EMG sensors were placed according to the guidelines reported by Barbero et al. (2012).

#### 2.2.1. MI group

MI group received MI-BCI rehabilitation training and routine rehabilitation training.

(1) MI-BCI rehabilitation training: The patient sat on a comfortable chair, 70 cm away from the monitor, the visual interface was located in the center of the 22 inch LCD, the resolution was 1920 × 1080 pixels, the screen refresh rate was 60 Hz, and the EEG sampling frequency was 1000 Hz (the electrodes were arranged according to the International 10–20 Standard Guide System). After sitting down, the patient began rehabilitation. The patient was relaxed and looked directly at the monitor. Based on the rehabilitation process of stroke patients, we built a set of MI-BCI visual and auditory stimulation systems using E-Prime 3.0 software (which can present a combination of multiple stimuli). Play a set daily motion animation of the upper limbs on the screen (including but not limited to grasping, placing, lifting, lowering, rotating, and other actions). The asynchronous amplitude (SOA) at the beginning of stimulation was set to 5 s to ensure that the patient understood the visual stimulation. This 5 s includes displaying a slow-moving image for 3.5 s and then masking the image for 1.5 s. One process included 12 image appearance processes, and the patients were allowed to rest for 1 min between processes. Complete rehabilitation training was repeated the whole process 50 times. During rehabilitation, the EEG acquisition system recognizes the signals of patients. The EEG signals were then converted into motion commands. The motor command controls the myoelectric stimulation system to stimulate the limb muscles of the patient. In the entire process of rehabilitation treatment, professional rehabilitation physiotherapists assist patients in completing their movements. During the rehabilitation process, the patient tries to avoid non-subjective movements (including, but not limited to, itching, coughing, yawning, blinking, etc.). The patient will be treated for 3 weeks, 5 days a week, for 45–60 m a day.
(2) Routine rehabilitation training, balance training, standing training, standing and sitting balance training, center of gravity transfer training, bed and chair transfer training, walking training, brace and auxiliary Walker training, and daily living ability training (grooming, mobility, dressing, and toilet). Physiotherapists will adjust the rehabilitation plan in a timely manner according to the patient’s condition, and evaluate the training effect.

#### 2.2.2. Control group

The control group only completed routine rehabilitation training. Training time, frequency, and content were the same as those in the MI group.

### 2.3. Functional assessment

After 21 days of rehabilitation training, the staff assessed the patients’ exercise abilities and physical status. All assessments were performed by the same trained staff who were blinded to the patient’s rehabilitation.

(1) FMA motor function score: The fugl-meyer assessment (FMA) was used to assess the upper limb motor function of patients in the MI and control groups, ignoring the upper limb sensory, passive joint mobility and pain sections of the table, and the reflexes section; only the 33 items in the upper limb section were used for this assessment, with a total score of 0–2 for individual items and 0–66 for the total score, based on 34 points for shoulder and elbow, 22 points for wrist and hand, and 10 points for motor coordination, with higher overall scores indicating better upper limb motor function.
(2) MAS motor function score: The motor assessment scale (MAS) was used to assess the comprehensive motor function and muscle tension of the body, which can effectively observe the curative effect and recovery of sensory movement on the affected side, ignoring the five functions of posture change and walking in the scale. This assessment used only four items: upper limb function, hand function, and general muscle tension. The individual score was 0–6 points, and the total score was 0–24 points. A higher score indicated better comprehensive motor function of the upper limb.
(3) Hyperdense middle cerebral artery sign (HMCAS): On non-contrast CT (NCCT) examination, the diseased cerebral artery presents a high-density signal compared to the contralateral one. It has been suggested that cerebral artery occlusion occurs in acute cerebral infarction in the cerebral artery-supplying area. NCCT can distinguish between cerebral hemorrhage and cerebral ischemia based on density changes in the visible vascular running area. A high density may lead to acute thrombosis, resulting in slow blood flow, stagnation, and even arterial occlusion.
(4) Clinical efficacy: Neurological deficit scores of Chinese patients with stroke were used as the criteria for determining efficacy. The functional impairment scores included the FMA, MAS, and HMCAS. When two of the three scores were met, patients were included at this level. Full rehabilitation: disability level 0 with 91% to 100% reduction in functional impairment score; significant rehabilitation: disability level 1–2 with 46% to 90% reduction in functional impairment score; general rehabilitation: disability level 3–4 with 18% to 45% reduction in functional impairment score; no change: no improvement in disease; deterioration (including death): increased functional impairment score compared to pre-rehabilitation. After rehabilitation training, general rehabilitation, significant rehabilitation, and full rehabilitation can be considered effective rehabilitation; no change or deterioration can be considered invalid rehabilitation.
(5) Brain topographic map: A plane graph of the spherical scalp represented by different colors of power values in each frequency band of the brain wave. When a certain region of the cerebral cortex is activated by various factors, the blood flow and metabolic activity in this region change with an increase in the activation intensity. As the stimulus increases, the effect of brain information modulation decreases and the amplitude of the EEG signal in the alpha and beta frequency bands decreases, which is called event-related desynchronization (ERD). That is, the event-induced spectral component was lower than the spontaneous spectral value, which was identified as negative in event-related spectral perturbation (ERSP). In contrast, the effect of information modulation in the brain is improved in the resting state, and the phenomenon of EEG signal spectrum amplitude is called event-related synchronization (ERS), which indicates that the event-induced EEG spectrum component is higher than the spontaneous EEG spectrum value, and it is identified as positive in ERSP.

### 2.4. Statistical analysis

Statistical software (SPSS 26.0) was used for the data analysis. The difference was considered statistically significant when the bilateral *p*-value was <0.05. The Shapiro–Wilk test was used to test the normality of quantitative data. (x ± s) was used to represent the measurement data conforming to the normal distribution. Qualitative data were explained by the number of cases and the component ratio (n [%]). The *t*-test was used to compare differences in continuous variables. χ2 test and comparison of differences in categorical variables.

## 3. Results and discussion

### 3.1. Comparison of patients

As shown in Table 1, in the MI group, there were 8 males and 12 females; the age range was 40–72 years, with a mean of (61.5 ± 3.8) years; the duration of illness was 14–38 days, with a mean of (20.1 ± 2.2) days. In the control group, there were 9 males and 11 females; the age range was 38–67 years, with a mean of (61.0 ± 3.7) years; the duration of illness was 16–40 days, with a mean of (20.6 ± 2.4) days. The differences between the MI and control groups were not statistically significant (*P* > 0.05).

**TABLE 1:** Basic patient information.

Information		MI group	Control group	*P*-values
Gender	Male	8	9	
	Female	12	11	
Age		61.5 ± 3.8	61.0 ± 3.7	0.90
Course of disease (day)		20.1 ± 2.2	20.6 ± 2.4	0.31
Affected side	Left	9	8	0.46
	Right	11	12	
Cerebral vascular disease	Yes	20	18	0.87
	No	0	2	
Coronary heart disease	Yes	17	14	0.68
	No	3	6	
Diabetes	Yes	6	7	0.74
	No	14	13	
History of hypertension	Yes	19	18	0.87
	No	1	2	
Hyperlipidemia	Yes	5	3	0.69
	No	15	17	
Drinking	Yes	6	7	0.75
	No	14	13	
Exercise habits	3 times a week or more	3	6	0.83
	Twice a week or less	10	10	
	None	7	4	
Total FMA scores		18.50 ± 6.46	18.70 ± 6.23	0.35
FMA shoulder and elbow scores		9.80 ± 3.51	9.50 ± 3.25	0.27
FMA wrist scores		5.65 ± 2.15	6.10 ± 2.14	0.15
MAS scores		6.00 ± 2.22	5.95 ± 2.68	0.24
Brain NCCT		83.65 ± 5.93	82.45 ± 5.70	0.79

### 3.2. Comparison of affected side FMA scores between the MI and control group

As shown in Table 2, after rehabilitation training, the total FMA score, shoulder and elbow score and wrist score of all patients improved significantly, and the differences were statistically significant (*P* < 0.05). All differences in the FMA scores were significantly higher in the MI group than in the control group after rehabilitation.

**TABLE 2:** Comparison of total FMA scores between the MI and control group.

Total FMA scores					
Groups	Before rehabilitation training	After rehabilitation training	Difference	*t*	*p*
MI group	18.50 ± 6.46	40.80 ± 14.92	16.70 ± 12.79	−3.141	0.003
Control group	18.70 ± 6.23	30.15 ± 15.06	5.34 ± 10.48	−4.759	0.004
Difference	1.31 ± 0.73	11.45 ± 6.90			
*p*	0.35	0.003			
**FMA shoulder and elbow scores**					
MI group	9.80 ± 3.51	20.85 ± 7.76	12.56 ± 6.37	−3.078	0.004
Control group	9.50 ± 3.25	15.30 ± 7.77	2.45 ± 7.91	−2.527	0.005
Difference	0.83 ± 0.51	6.21 ± 2.12			
*p*	0.27	0.005			
**FMA wrist scores**					
MI group	5.65 ± 2.15	13.00 ± 5.11	11.01 ± 3.48	−2.938	0.006
Control group	6.10 ± 2.14	9.75 ± 5.15	3.36 ± 5.79	−2.271	0.007
Difference	0.42 ± 0.12	4.27 ± 1.43			
*p*	0.15	0.007			

### 3.3. Comparison of affected side MAS score of between the MI and control group

As shown in Table 3, after rehabilitation training, the MAS scores of both groups were higher than before treatment, and the MAS scores of the MI group were significantly higher than those of the control group after treatment, and the difference was statistically significant (*P* < 0.05).

**TABLE 3:** Comparison of MAS scores on the affected side between the MI and control group.

Groups	Before rehabilitation training	After rehabilitation training	Difference	*t*	*p*
MI group	6.00 ± 2.22	13.6 ± 5.18	3.62 ± 2.48	-3.50	0.001
Control group	5.95 ± 2.68	10.75 ± 5.51	1.85 ± 2.89	0.157	0.002
Difference	0.59 ± 0.13	3.98 ± 1.28			
*p*	0.24	0.006			

### 3.4. Comparison of brain status between the MI and control group

As shown in Table 4, hyperdense middle cerebral artery sign is an indirect sign of cerebral infarction caused by occlusion of the cerebral artery. The high-density component represents blood clots, thrombi, or emboli in the cerebral artery lumen. The CT value of flowing blood is approximately 40Hu, which is linearly correlated with the hemoglobin concentration. Before rehabilitation treatment, the arterial high-density sign of NCCT was relatively high, and after rehabilitation training treatment, the NCCT arterial high-density sign values of the two groups were lower than those before rehabilitation treatment, and those of the MI group were lower than those of the control group, and the difference was statistically significant (*P* < 0.05).

**TABLE 4:** Comparison of the affected brain NCCT between the MI and control group.

Groups	Before rehabilitation training	After rehabilitation training	Difference	*t*	*p*
MI group	83.65 ± 5.93	60.70 ± 11.71	21.94 ± 2.37	3.216	0.003
Control group	82.45 ± 5.70	72.10 ± 13.21	17.86 ± 3.55	-10.99	0.003
Difference	3.27 ± 0.97	15.76 ± 3.21			
*p*	0.79	0.002			

### 3.5. Comparison of clinical efficacy between the MI and control group

As shown in Table 5, full rehabilitation, significant rehabilitation, and general rehabilitation can be considered effective rehabilitation; no change or deterioration can be considered invalid rehabilitation. The total effective rate of the MI group was 95% (19/20), which was higher than that of the control group (75%, 15/20).

**TABLE 5:** Comparison of the clinical efficacy between the MI and control group.

Clinical efficacy	MI group	Control group
Full rehabilitation	3 (15%)	1 (5%)
Significant rehabilitation	10 (50%)	7 (35%)
General rehabilitation	6 (30%)	7 (35%)
No change	1 (5%)	5 (25%)
Deterioration	0	0
Effective rehabilitation	19 (95%)	15 (75%)

### 3.6. Comparison of brain topographic between the MI and control group

The average power spectral density (PSD) of each channel was calculated according to the spatial spectral characteristics of EEG signals under tactile stimulation feedback. Finally, the PSD value was mapped to the topographic map to determine the spatial spectral characteristics of brain oscillations. The event-related spectral perturbation power of all subjects in typical frequency bands of 8–30 Hz (alpha:8–13 Hz, beta:14–29 Hz) (Pfurtscheller and Da Silva, 1999; Graimann et al., 2002) was extracted, and a brain topographic map was drawn.

**FIGURE 2:**
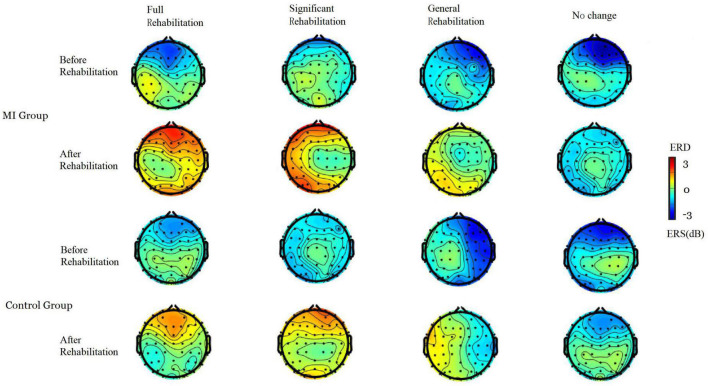
Brain topographic of MI group and control group.

As shown in Figure 2, after 3 weeks of training, compared with passive routine rehabilitation, the rehabilitation process with MI-BCI, EMG stimulation, and feedback had significantly higher ERD at–8–30 Hz (alpha band and beta band). This phenomenon indicates that the brain actively participates in physical activity. By comparing PSD, the ERD characteristic sensory pattern of motor-related areas in the full rehabilitation MI group was significantly enhanced, but the regularity of enhancement was different, and the power distribution area was relatively scattered. In addition, it was found that during motor imagery, the PSD topographic maps of both limbs were similar and the bilateral sensory cortex areas were activated. Comparing the brain topography of the two groups before and after rehabilitation, the full rehabilitation patients had the strongest response to the MI-BCI stimulation; the significant rehabilitation patients had a more obvious response to the stimulation, the general rehabilitation patients had no obvious response to the stimulation, and the no change patients had no response to the stimulation. Compared with the two groups, the response of the MI group patients with full rehabilitation and significant rehabilitation to MI-BCI stimulation was completely stronger than that of the control group patients. However, the responses of the two groups of general rehabilitation and no change in patients to stimulation were similar to those of patients, and there was no significant difference. This shows that the active and participatory MI-BCI and FES upper limb rehabilitation system can effectively mobilize patients’ multi-sensors to participate in rehabilitation treatment at the same time, and the rehabilitation effect is significantly better than that of passive routine treatment.

As shown in Figure 2, some subjects (full rehabilitation and significant rehabilitation) showed obvious changes in the distribution of brain topological power induced by rehabilitation training. General Rehabilitation changed, but some patients did not respond to rehabilitation. This also confirmed our view that the MI-BCI rehabilitation system can promote the rehabilitation of stroke patients, however, not all patients have a therapeutic effect. Due to the special condition of the brain of stroke patients, we believe that the patient’s attention concentration may affect the use of BCI devices, resulting in some patients being unable to use BCI in the early stage of rehabilitation treatment. Poor motor function, speech disorder, and spasm during rehabilitation may be adverse factors affecting the rehabilitation of upper-limb motor function in stroke patients.

### 3.7. Discussion

This study used multisensory MI-BCI and FES feedback to verify the neural plasticity of stroke patients (Wolpaw, 2007; Biasiucci et al., 2018; Cervera et al., 2018). Exploring the related brain electrical activity provides roadmaps for brain research and clinical diagnosis. EEG studies have revealed secrets of brain cognitive functions and neural disorders (Luo et al., 2022). Neuromotor rehabilitation promotes neuroplasticity, favoring functional recovery of the ipsilesional hemisphere and activation of anatomically and functionally related brain areas in both hemispheres to compensate for damaged tissue (Tavazzi et al., 2022). Based on brain-machine- muscle-brain closed-loop feedback, a closed-loop neural feedback path is constructed between patients and rehabilitation equipment (Ang et al., 2015). Many studies have shown that BCI combined with EMG and exoskeletons can effectively promote rehabilitation of limb motor function. EEG studies have shown the importance of physical activity in promoting learning, and the effects of inactivity or microgravity on cortical reorganization to cope with absent or altered sensorimotor stimuli. Changes in power bands in different cortical areas occur with fatigue and in response to training stimuli, leading to learning processes (Manganotti et al., 2022). Biasiucci et al. (2018) built a BCI-FES upper-limb rehabilitation system for stroke. According to the observation after 6–12 months of follow-up, Biasiucci used the Fugl-Meyer clinical scale to verify the effectiveness of the BCI-FES rehabilitation system in restoring motor function and stimulating nerve rehabilitation (Biasiucci et al., 2018). For the analysis of EEG, previous studies have shown that patients with stroke can elicit ERD/ERS during motor imagining (Pfurtscheller and Aranibar, 1979; Neuper et al., 2006). In this study, ERD and ERS were used to characterize the change in feature level induced by rehabilitation training. For the patients with effective rehabilitation (34/40), the FMA, MAS, HMCAS, and PSD scores in the MI group were significantly higher than those in the control group, which showed that the MI-BCI upper limb rehabilitation system based on closed-loop feedback could promote the recovery of the motor function of the affected side and induce changes in the brain nerves. The results validated that after MI training, the activation degree of the motor cortex and the connection level of the related cortical functional network in patients with stroke were significantly improved (Mueller-Putz et al., 2014; Delorme et al., 2019; Wang et al., 2019). In this study, active evoked stimulation of the brain, muscles, and nerves was achieved through motor imagination and FES. For routine rehabilitation training, patients’ limb movement rehabilitation is mainly passive muscle movement and nerve stimulation, ignoring the command role of the brain in the human body; therefore, the recovery efficiency is far lower than that of active induction. There was a problem with the length of the rehabilitation. The MI group used two rehabilitation methods, leading to a longer period of rehabilitation than the control group, which would affect the scoring. The experiment did not remove this influence; therefore, this will be a matter of our follow-up research.

## 4. Conclusion

For upper limb motor function rehabilitation in stroke patients, BCI combined with external devices may be more effective than conventional rehabilitation strategies, but there is a lack of comprehensive evaluation of motor function rehabilitation in patients. In this study, MI-BCI was combined with FES to perform multisensory stimulus feedback, including visual, auditory, and tactile stimuli, and an upper limb rehabilitation system based on MI-BCI was established. We used the clinical scales FMA, MAS, and NCCT to comprehensively evaluate the rehabilitation effect of MI-BCI. Clinical scale results showed that FMA, MAS, and NCCT scores significantly improved after Mi-BCI rehabilitation treatment, and the scores of patients in the MI group were significantly higher than those in the control group. These results indicate that MI-BCI rehabilitation training can effectively improve motor function in stroke patients, which is consistent with the results of previous studies. In this study, we used an EEG-based brain topographic map to comprehensively study the brain activity of stroke patients after rehabilitation therapy with MI-BCI. According to the analysis of the brain topographic map, the brain ERD of patients with full rehabilitation and significant rehabilitation in the MI group was significantly higher than that in the control group, however, there was no significant difference in ERD between patients with general rehabilitation and no change in the two groups, indicating that if patients actively participate in the rehabilitation of MI-BCI, the feedback of myoelectric stimulation induced by FES could effectively promote nerve remodeling and improve the effect of rehabilitation. The total effective rate of the MI group was 95% (19/20), which was higher than that of the control group (75%, 15/20). The results of this study prove that MI-BCI rehabilitation therapy is both effective and feasible. However, there was one patient in the MI group whose condition did not change, and three patients were excluded because the accuracy of EEG collection was lower than the standard, which was probably caused by adverse factors such as brain damage, poor motor function, speech disorders, and spasms in stroke patients.

## Data availability statement

The raw data supporting the conclusions of this article will be made available by the authors, without undue reservation.

## Ethics statement

The studies involving human participants were reviewed and approved by the Biomedical Ethics Committee of Hebei University of Technology. The patients/participants provided their written informed consent to participate in this study.

## Author contributions

WL and CL conceived the study. WL and JL collected the data and wrote the manuscript. JL conducted the experiments. XZ performed the EEG data processing. CL reviewed and edited the manuscript. All authors have reviewed the manuscript, agreed to its submission, read, and approved the final manuscript.
